# Role of Interface Design: A Comparison of Different Online Learning System Designs

**DOI:** 10.3389/fpsyg.2021.681756

**Published:** 2021-08-31

**Authors:** Aiqin Shi, Faren Huo, Dongnan Han

**Affiliations:** ^1^College of Arts and Design, Nanjing Forestry University, Nanjing, China; ^2^Pan Tianshou College of Architecture, Arts and Design, Ningbo University, Ningbo, China; ^3^College of Arts and Design, Inner Mongolia University of Science and Technology, Baotou, China

**Keywords:** user experience, older adults, online system learning, navigation design, online education, layout design

## Abstract

Mobile devices are becoming an indispensable part of the daily lives and learning habits of older adults with the easy access of the Internet. It enhances the connection between old users and online education, which supplies an approach to cultivate them with innovative concepts and entrepreneurship of education. However, the complicated navigation of information systems (IS) confuses older adults, and gets them disoriented in searches of information, in addition, to influencing online activities for older adults. This study aims to investigate what kind of navigation of IS is suitable for older adults. A 2 × 2 (2 factors, 2 levels) mixed experimental design was employed. The two factors were, respectively, cognitive load (CL) and navigation structure (NS). A sample of 40 older participants (mean age = 64.37, SD = 4.03) performed online learning tasks in terms of innovative concept using linear hierarchical or mixed NSs under different time pressures. The results showed that linear hierarchical navigation is more appropriate for the elderly when learning and generating innovative concepts on smartphones, as the interaction between CL and NS exists. Overall, the findings combined suggest that the linear hierarchical NS, compared to mixed hierarchical navigation, obtained better usability in terms of task efficiency, CL, and subjective ratings. The findings can provide theoretical support for designers to design and develop mobile websites for older adults.

## Introduction

With easy access to the Internet, an increasing number of older adults are using mobile phones for information searches. Older adults tend to be limited in activities due to capability impairment or loss of memory or cognitive, motor, and vision abilities. They experience more usability challenges than younger adults (Vines et al., 2015; Lim and Han, 2020).

Numerous studies have focused on usability guidelines (Hoehle et al., 2016) and heuristic evaluation (Mi et al., 2014; Troussas et al., 2020) to create user-friendly mobile applications. However, previous studies have explored usability issues regarding visual and haptic features such as size, space, color, and method of touch (Jung and Jang, 2015). Moreover, most existing studies have examined navigation usability of feature phones or in the desktop context (Dianat et al., 2019). Since 2007, navigation, a means of information retrieval (Rist, 2009; Garrett, 2010), has become increasingly complicated with the emergence of touch screens. New navigation patterns have resulted in greater cognitive load (CL) and usability issues (Li and Luximon, 2016, 2018, 2019). Studies have not thoroughly explored navigation issues that require more cognitive and perceptual processing (Petrovčič et al., 2017).

Navigation and time pressure can help users accomplish tasks and accelerate decision making (Khoo and Mosier, 2008; Strong, 2009; Garrett, 2010), thereby enhancing user experience (UX). Paas and Van Merriënboer (1994) indicated that excess time pressure decreases information search efficiency. However, the effects of navigation structure (NS) and time pressure on UX for older adults with declining capabilities remain unsettled (Punchoojit and Hongwarittorrn, 2017). Accordingly, this study examined the effects of NS and CL on usability and affective experience with web browsing on smartphones for older adults.

## Related Work

### Navigation Structure

Navigation structure is intended to provide easy access to significant information. It determines the order of access to information units and thus the effort required to navigate from the entry point to a specific unit. An effective NS indicates where users are and where they can proceed next (Chen et al., 2006). Several studies have explored the effects of NS on desktop usability (Fang and Holsapple, 2007). Fang and Holsapple (2011) confirmed that for both simple and relatively complex knowledge acquisition tasks, a usage-oriented hierarchy or a combined hierarchy has significantly higher performance than a subject-oriented hierarchy. Calisir and Gurel (2003) examined the influence of text structure (traditional linear text, hierarchical, and mixed hypertext) and learner’s prior knowledge on reading comprehension, browsing, and perceived control. They revealed that hierarchical hypertext is most suitable on a PC for individuals without relevant knowledge of experimental materials.

Of the limited studies on mobile navigation, most studies on usability have focused on feature phones. Kim et al. (2011) explored menu structures, item categorizations, task complexity, and menu patterns. Lin (2003) illustrated that in terms of browsing and navigation efficiency, hypertext with a hierarchical topology was superior to its referential counterpart for older adults. Parush and Yuviler-Gavish (2004) investigated the appropriate hierarchy depth of websites. They indicated that a broad NS was superior to a deep structure for cellular phones. Older adults experienced increased working memory load and disorientation when faced with deep structures.

Considering navigation usability on mobile phones entirely changed with the emergence of smartphones, Punchoojit and Hongwarittorrn (2017) observed notable differences between mobile phones and desktops in terms of the lack of tactile feedback, ubiquity, limited screen size, and the small virtual keys of phones. They claimed that older adults faced difficulties in information searches; however, this fact remains underexplored. Therefore, the effectiveness of navigation patterns on smartphones for older adults should be further examined.

### Cognitive Load

Cognitive load refers to the total amount of cognitive resources needed to process information during the task. Ayres (2006) claimed it was the burden placed on the working memory during problem solving and learning. Researchers argued that the overloading working memory tended to inhibit learning. When cognitive overload is avoided, instructional procedures would be the most effective. Sweller (1999) suggested that with the increase of CL, task performance first improves; when CL increased to a certain extent, task performance decreased; too low or too high cognitive load (HCL) would reduce task performance.

With respect to navigation processing, previous literature on CL focused extensively on how to evaluate CL. For instance, Sayago and Blat (2010) studied how the elderly use email and illustrated that CL related to digital tasks was the most influential accessibility barrier. Rapp et al. (2003) reviewed relevant findings from cognitive research on text comprehension, memory, and spatial cognition with the aim of describing how these concepts apply to the design and functionality of digital libraries. Later, Destefano and Lefevre (2007) evaluated the effects of hypertext features on cognitive processing during text navigation and comprehension; they predicted an increase of CL and impairment of learning for hypertexts with a higher number of links per page. Madrid et al. (2009) tested DeStefano and LeFevre’s predictions and claimed there was a benefit of using link suggestions for learning, but no effect of number of links on learning was found; compared to linear reading, extra cognitive resources were required in the process of choosing a link.

Many factors impact CL, such as prior knowledge, experience, working memory capacity, etc., and in general these factors need to be controlled in the study (Destefano and Lefevre, 2007). Task difficulty, complexity, and time pressure are commonly used technologies to control CL in previous studies (Paas and Van Merriënboer, 1994). Time pressure is a key influential factor in information search tasks. In the information behavior domain, impacts of time pressure in terms of decision making were widely studied (Landry, 2016; Tinghög et al., 2016). Therefore, we chose time pressure as the method to manipulate CL conditions in this study.

It should be noted that the literature on the effect of CL concentrated mainly on the users’ speed and accuracy. However, little research attempted to demonstrate the influence of CL on affective experience of older adults concerning mobile navigation behavior.

### User Experience

User experience has been interpreted from various perspectives (Hassenzahl and Tractinsky, 2006; Kim, 2015; Yang et al., 2019). The complexity of experience should be categorized into evaluative constructs such as usability and emotions (Pucillo and Cascini, 2014). UX can be categorized into practical and hedonic experiences (Hassenzahl, 2001). Practical experience refers to system usability, functional usefulness, and ease of use. Need fulfillment was clearly linked to hedonic quality perceptions but not as strongly to pragmatic quality and was a source of positive experience with interactive products and technologies (Hassenzahl et al., 2010).

Affect is a barometer of individuals’ psychological state, and life event is an essential prerequisite for daily affective experience (Berkowitz, 2003). Zautra et al. (2005) advanced the theory and methods of the dynamics of emotional life. They argued that researchers should simultaneously incorporate negative and positive dimensions of personality, life events, and psychological well-being. Information searching on mobile websites is a daily life event. According to Zautra et al. (2005), positive or negative affect can be observed during information searches.

Neilson’s heuristic measures have been widely used on mobile usability (Salgado and Freire, 2014). However, it mainly focused on practical experience such as ease of use, response speed, and friendly interface, which may not be adequate for older adults (Nielsen and Levy, 1994). Park et al. (2016) claimed that usability enables users to use products smoothly and that better hedonic experience retains users as well as predicts users’ behavioral intentions. UX goals are subjective qualities and are concerned with users’ perceptions of products (Punchoojit and Hongwarittorrn, 2017). Therefore, understanding older people’s navigation preference and usability issues on smartphones and exploring factors determining variation in their affective experience are essential.

### The Present Work

Above all, most mobile navigation designs are based on desktop paradigms, but desktop designs do not completely fit the mobile context. Furthermore, CL of web browsing for older adults is more easily overloaded, thereby decreasing UX. Therefore, this study addressed the following research questions:

1. Do NS and CL have significant effects on older users’ affective experience, usability, and task performance?
2. Which kind of NS is best for older adults?

We conducted an experimental study using the National Digital Library of China (NDLC) website on smartphones to investigate the effects of NS and CL on older users’ affective experience with web browsing. The website has two versions, one with a linear hierarchical structure and one with a mixed hierarchical structure. The classic Positive Affect Negative Affect Scale (PANAS), which has demonstrated high reliability (Tellegen, 1988), was employed to measure affective experience. The classic System Usability Scale (SUS) comprising 10 questions (Brooke, 1996) was used to measure usability. We hypothesized that NS and CL significantly impact affective experience, usability, and task performance and that a linear hierarchical NS is more suitable for older adults.

## Method

### Design

A Huawei Nova 2 smartphone was employed with a resolution of 2,160 × 1,080 to conduct the usability and UX testing in a lab. Screen brightness and environmental light over the experiment were unified. The NDSL websites that included two versions of NSs mentioned above were utilized in the usability and UX testing.

**FIGURE 1:**
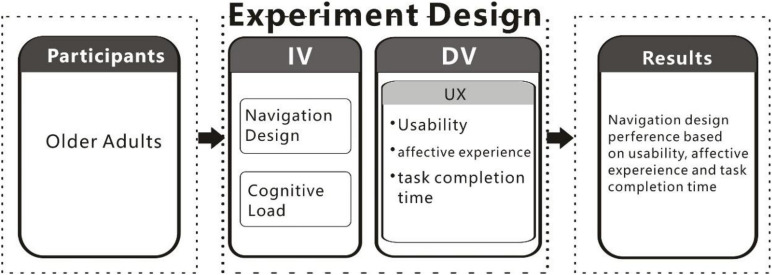
Experiment design.

A 2 × 2 (2 factors, 2 levels) mixed experiment was designed. The two factors were, respectively, CL and NS. CL is a between-subjects factor with high and low levels using time pressure control technology. NS is a within-subjects factor including two experimental conditions: (1) A-linear hierarchical structure; (2) B-mixed hierarchical structure (see Figure 1), corresponding to the two versions of the NDLC website. As complementary data, an eye tracker was used. The experiment design is shown in Figure 1.

### Participants

A total of 40 participants were recruited from a university for the aged. The participants’ ages ranged from 60 to 70 (*M* = 64.37, SD = 4.03). Eighteen participants were male and the others were female. All participants graduated from high school or above. Each participant spent more than 3 h a day reading on a mobile phone on average. All participants were corrected to a normal vision and right-handed. All participants gave informed consent before the experiment and were paid for participation.

According to the mixed experimental design, 40 participants were divided into two groups on average [HCL group and low cognitive load (LCL) group]. Each group consisted of 20 participants. Every participant performed two sets of experimental tasks, respectively, in linear hierarchical navigation and mixed NSs.

## Materials

**FIGURE 2:**
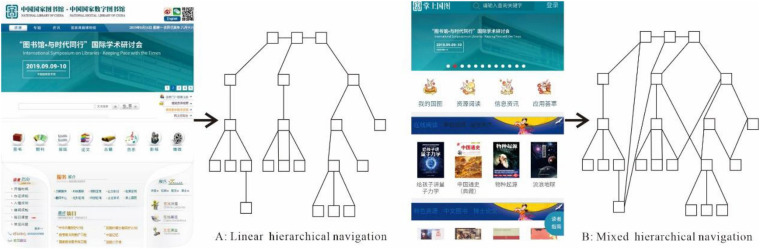
Two versions of navigation structure of NDLC websites.

The two experimental versions of the NDSL website used in this study were based on the studies of Calisir and Gurel (2003). These materials were typically adapted to be under two conditions (linear hierarchical structure, and mixed hierarchical structure) on smartphones. In the two conditions, the information was identical. The linear hierarchical navigation was linear homepage with hierarchy. It formed a strict hierarchy. Participants moved through the homepage by clicking words on the screen. A number of cross-referential links in the hierarchical homepage created the mixed hierarchical homepage. Users were allowed to move through the homepage without following a strict hierarchy, as shown in Figure 2.

### Procedure

Before starting the study, a pilot test was carried out with twenty 60–70 year-old participants who had similar characteristics to the target participants. The aim of this study was to adjust time pressure and measure CL. All data from these users were not included in the final sample.

Ten users were invited to perform A/B testing randomly (see more in Table 1), and the interval between two tests was 30 min. Four users conducted from A to B, six users conducted from B to A. Then, the average completion time was computed (*M* = 240, SD = 20). Later, based on the above average time, another five users performed A or B tasks randomly within 300 s (adding 3 SD, namely: 60 s), the other five users performed A or B tasks randomly within 180 s (subtracting 3 SD, namely: 60 s). Right after they completed the predefined tasks, CL value was measured using the PAAS scale (Paas and Van Merriënboer, 1994). The CL was approximately 3.3 and 6.1 points, respectively. Therefore, conditions for the small- and big-time pressure were concluded in line with the low and HCL. In this study, the corresponding task time constraints were 300 and 180 s, beyond the limit of time would be considered as a failure.

**TABLE 1:** Experimental tasks.

No.	Tasks of linear hierarchical navigation	Tasks of mixed navigation
1	Find book ≪Shan Hai Jing≫ and add to the bookmark.	Find book ≪Four Generations≫ and join a bookcase.
2	Check the ranking list, find book ≪Records of the States in the Eastern Zhou Dynasty≫, open the book.	Check the ranking list, find book ≪Beijing, Beijing≫, open the book.
3	Find holiday (the Mid-Autumn festival and National Day) opening arrangement of NDLC, 2017	Find the latest announcement, “NDLC” website online announcement.
4	Find the lecture by Xin Deyong: Finding ≪YanRan Mountain Inscription≫ – discussion of the academic value of the discovery, please name the lecture time and venue.	Find the lecture by Wu Wenling: Ancient China recorded by unearthed silk, please name the lecture time and venue.
5	View the online exhibition “Ode to the motherland.”	View the online exhibition “Reunion of one hundred children.”

After the pilot study, we carried out the formal tests. Participants took a rest for 3–5 min after entering the lab. They listened to the experimental introductions and signed a consent form. This study implemented a pre-test-post-test design to investigate affective experience variation using the PANAS scale, and a post-test was delivered to obtain usability information using the SUS scale. All participants went through both experimental conditions randomly: A = website with linear hierarchical navigation and B = website with mixed hierarchical navigation. Prior to the start of each experimental version, participants filled out the PANAS scale, and affect was recorded as later reference standard. After a version of the test was finished, participants filled in PANAS scale again, and then filled in the SUS scale. The interval for the two versions of the tests was 30 min. The test time for each participant lasted approximately 40 to 50 min.

### Experimental Tasks

All participants performed tasks of the same quantity and similar contents in the two versions of the NDLC website, as shown in Table 1. All tasks were required to finish randomly within the given time. Completion time of each task was recorded. To eliminate the effects of the order, each participant chose the experimental version randomly. In the LCL group, the number of participants going from A to B interface was the same as the number of participants going from B interface to A interface; in the HCL group, participants going from A to B interface were 8 people, while participants going from B interface to A interface, were a total of 12 people.

### Data Acquisition

Two sets of data were collected: the objective data and subjective data. Objective data referred to the behavior data during the experiment including the completion time of each task and success rates. To accurately measure the completion time of each task, Dkablis glasses were used with a sample rate of 60 Hz. All objective data were analyzed using D-Lab data analysis system. Subjective data referred to affective experience and usability data, respectively, from PANAS scale and SUS scale, which were analyzed using Stata14.0.

## Results

### Behavioral Results

Completion time and success rates were averaged separately, the results were shown in Table 2. Average time for task 1 was the longest, while average times for task 2 and task 3 were similar and a little shorter than task 1. Task 4 and task 5 were finished in the shortest time.

**TABLE 2:** Mean value of completion time and success rate of each task.

	Task 1	Task 2	Task 3	Task 4	Task 5
Mean (SD)	66 s (17.5 s)	42 s (15.7 s)	42 s (16.0 s)	34 s (10.1 s)	33 s (11.3 s)
Success rate (%)	100	100	100	70	55

Mixed ANOVAs were conducted using task time as dependent variable. The result showed that the interaction effects between CL and NS were remarkable on completion time (*F* = 4.88, *p* = 0.035), but the main effects of CL and NS on completion time of task 1 were not significant (*p* > 0.05). NS had significant main effects on the completion time of task 3 (*F* = 4.12, *p* = 0.05) and task 5 (*F* = 6.5, *p* = 0.02). In the current experiment, the completion time of task 1 only changed with the simultaneous changes of CL and NS, and was not affected by CL or navigation separately. Completion time of task 3 and task 5 changed with the change of NS significantly.

Simple effect analysis of interaction effects between CL and NSs on the completion time of task 1 was carried out. For fixed linear hierarchical navigation, completion time decreased with the increase of CL, which indicated that time pressure in linear hierarchical navigation helped older readers to complete tasks faster. For fixed mixed navigation, completion time was extended while CL changed from low to high, which showed that completion time for older readers in mixed navigation was greatly influenced by time pressure. Time pressure played a role in the extension of task completion time.

### Affective Experience Results

Affective experience variation was examined using the method of post-test value minus pre-test value. Before commencing each test, the basic affective experience of participants was measured. Pre-test values of positive affect (PA1) and negative affect (NA1) were acquired. After finishing the test of each version, the post-test value of affective experience was measured immediately. The results were PA2 and NA2. The score difference of two positive affects was the variation of positive affective experience; the algorithm of negative affective experience was the same as positive affective experience. The statistical results were as shown in Table 3.

**TABLE 3:** Affective experience measurement results in different conditions.

Condition	Statistics	PA1	NA1	PA2	NA2	ΔPA	ΔNA
High cognitive load	*M*	3.47	1.27	3.9	1.03	0.48	–0.19
Linear hierarchical navigation	SD	0.83	0.66	0.79	0.07	0.71	0.34
High cognitive load	*M*	3.47	1.27	2.59	1.56	–0.89	0.29
Mixed navigation	SD	0.83	0.66	0.94	0.54	0.69	0.54
Low cognitive load	*M*	3.47	1.27	3.16	0.73	–0.31	–0.54
Linear hierarchical navigation	SD	0.83	0.66	1.14	0.07	0.54	0.65
Low cognitive load	*M*	3.47	1.27	3.52	1.16	0.05	–0.11
Mixed navigation	SD	0.83	0.66	1.01	0.32	0.78	0.52

**FIGURE 3:**
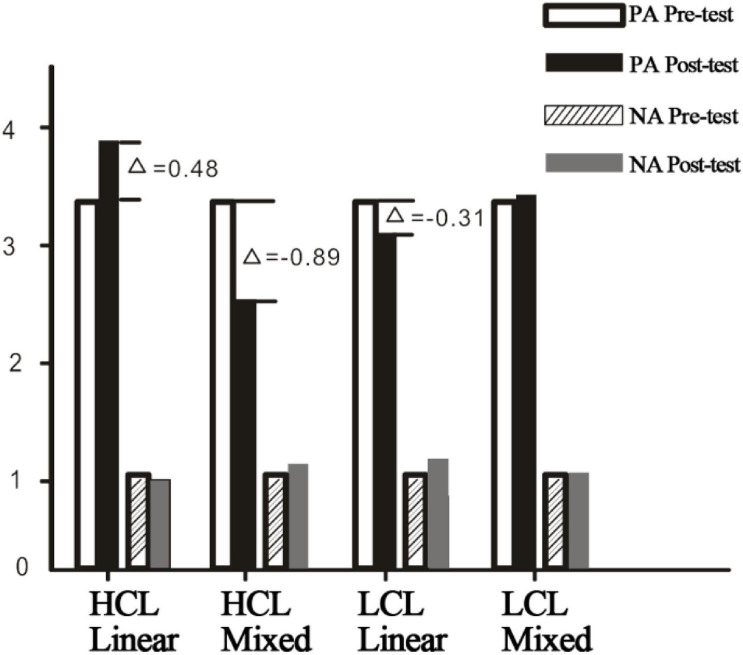
Comparison of affective experience variation.

Figure 3 shows the visualization of affective experience data. The result showed that the mean value of the reader’s positive affect was increased in HCL and linear hierarchical navigation conditions (ΔPA = 0.48), while the negative experience was reduced (ΔPA = 0.19). High cognitive load and mixed navigation as well as LCL and linear navigation reduced the reader’s positive affective experience, HCL and mixed navigation greatly reduced the positive affective experience (ΔPA = −0.89), but also increased the negative affect (ΔPA = 0.11). LCL and mixed NS had little influence on positive and negative affective experiences.

To further examine how CL and NS impact affective experience, mixed ANOVAs were carried out using CL and NS as independent variables, and positive affective variation as a dependent variable. The results showed that the effects of NS were marginally significant on positive affective variation (*F* = 3.88, *p* = 0.058), and the main effects of CL on positive affective variation were not significant (*F* = 0.18, *p* = 0.677), but the interaction effects of CL and navigational structure on positive affective variation were significant (*F* = 13.66, *p* < 0.001), which indicated that only when the two independent variables worked together will they significantly influence affective experience. After interaction effects were confirmed, simple effect analysis showed that when NS was fixed, positive affect in the linear navigation condition was elevated along with the increase of CL. Positive affect in the mixed navigation condition was decreased along with the increase of CL. In the fixed LCL condition, positive affective experiences in two NSs were similar, and the score of mixed navigation was slightly higher. In fixed HCL condition, affective experience differences were significant, positive affective experience in linear navigation was higher.

To further study how CL and NS influence changes in negative affective experience, mixed ANOVAs were conducted using negative affective experience variation as dependent variables, the results showed that effects of CL (*F* = 0.55, *p* = 0.47) and NSs (*F* = 2.2, *p* = 0.14) on negative affective experience variation were not significant, and no interaction effects were found in between (*F* = 0.02, *p* = 0.89).

### Usability Results

After completing the test tasks, the affective experience of participants was rated first, and then the product usability was rated. The statistical results in 4 test conditions were shown in Table 4.

**TABLE 4:** Usability scores in different conditions.

Condition	Statistics	SUS score
High cognitive load	*M*	90.31
Linear navigation	SD	5.89
High cognitive load	*M*	58.88
Mixed navigation	SD	22.78
Low cognitive load	*M*	79.06
Linear navigation	SD	16.74
Low cognitive load	*M*	71.88
Mixed navigation	SD	16.24

Usability scores in the linear navigation condition were slightly higher than in the mixed navigation condition, and usability scores in the low and high cognitive load were similar. Further mixed ANOVAs found that the CL did not significantly affect usability scores (*F* = 0.07, *p* = 0.79), but the NS affected the usability significantly (*F* = 7.99, *p* = 0.009), interaction effects of the CL and NS were not significant (*F* = 3.33, *p* = 0.079).

### Usability and Affective Experience

To examine if usability and affective experience have correlations, Pearson correlation coefficient analyses on usability scores, and positive and negative affective experience variation scores were carried out. The results showed that positive affective experience variation had significant correlations to negative affective experience variation (*r* = −0.493, *p* = 0.005), but positive affective experience variation had no significant correlations to usability (*r* = 0.323, *p* = 0.076), negative affective experience variation had no significant correlations to usability (*r* = −0.137, *p* = 0.462).

## Discussion

### Cognitive Load Had Little Influence on Task Performance, Affective Experience, and Usability

Behavioral results showed that CL had no significant effects on the completion time of five tasks, but significantly influenced the success rates of task four, and significantly impacted the success rates of on task five. Success rates of task four and task five in heavy time pressure conditions were lower than in small time pressure conditions, as shown in Table 5. Previous study reported that pressure would increase people’s information processing speed, and caused people to pay attention to the outline of things without in-depth analysis (Mannix, 2010), hence it was guessed time pressure would affect task completion time. The findings of this study were consistent with Allahverdipour et al.’s (2020) finding that CL significantly influenced the task efficiency for older adults. All participants in the current study used NDLC for the first time. In the big time pressure condition, the completion time of task 1 was much more than the other tasks. Table 2 demonstrated that follow-up tasks were improved gradually. This implied that participants consumed a lot of time learning the website at the early stage of tasks. The results showed that time pressure had no significant effects on complete time of task 1, but time pressure and NS had interaction effects, which indicated that two factors alone would not much affect completion time of task 1, only two factors working together would exert significant effects.

**TABLE 5:** Mixed ANOVAs results of task time.

Task 1	Behavioral data	MS	*F*	*p*
Cognitive load	Task time	136.13	0.48	0.49
Navigation structure	Task time	36.13	0.13	0.72
Cognitive load × Navigation structure	Task time	1378.13	4.88	0.035*
**Task 2**				
Cognitive load	Task time	81.28	0.35	0.56
Navigation structure	Task time	850.78	3.67	0.06
Cognitive load × Navigation structure	Task time	195.03	0.84	0.37
**Task 3**				
Cognitive load	Task time	96.56	0.40	0.53
Navigation structure	Task time	1005.72	4.12	0.050*
Cognitive load × Navigation structure	Task time	19.51	0.08	0.7796
**Task 4**				
Cognitive load	Task time	250.71	2.55	0.1,222
Navigation structure	Task time	57.57	0.59	0.45
Cognitive load × Navigation structure	Task time	0.91	0.01	0.92
**Task 5**				
Cognitive load	Task time	179.44	1.86	0.19
Navigation structure	Task time	618.21	6.40	0.02*
Cognitive load × Navigation structure	Task time	221.99	2.30	0.14

Note: * refers to p < 0.05.

Affective experience analysis revealed that effects of CL on positive affect and negative affective experience variation were not significant. Existing research suggested that too much pressure may cause unpleasant physical, affective, and cognitive, effects, and may also bring negative effects such as anxiety (Salas et al., 1996). The results in this study did not find that the CL had significant effects on affective experience variation, but found that interaction effects between CL and NS on affective experience variation existed, which indicated effects of CL on affective experience would work only when it worked with NS. Besides, simple effect analysis revealed that when time pressure was combined with mixed navigation, increasing time pressure cannot effectively accelerate the completion of task 1, yet it can arouse reverse inhibitory effects. For people in the LCL, the NS did not affect how positive they found the website. However, for people in the HCL, the NS did affect how positive they felt: when HCL people saw the linear hierarchical structure, their emotion improved, but when they saw the mixed structure, their emotion grew more negative.

Usability analysis results showed that CL had no significant effects on usability, which implied that readers did not attribute the failure to perform tasks to usability. Post-test interview results indicated that readers used NDLC with much confidence. They were already familiar with the product after performing tasks. In addition, they made a comprehensive evaluation of mobile websites.

In sum, CL significantly influenced success rates of tasks, and did not significantly affect usability. The effect of CL on success rates of task 4 and task 5 was significant, but the success rates reflected the effectiveness in usability. It had effects on completion time and affective experience of task 1 only when CL worked with NSs.

### Navigation Design Significantly Influenced Task Performance, Affective Experience, and Usability

The experimental results indicated that NS did not significantly affect success rates, but had remarkable effects on completion time of task 3 and task 5, and affected completion time of task 1 together with CL. Further analysis showed that completion time in linear hierarchical structure was less than in mixed NS, which indicated that the elderly in linear hierarchical navigation performed tasks more easily and efficiently. NS helps users to quickly find the required functions. Existing study showed that linear hierarchical structure performance was superior for elderly people to the hyperlinked navigation. Castilla et al. (2016) have made a test of email system navigation for the elderly, the elderly were asked to send and receive email through the linear hierarchical navigation or hyperlinked navigation, respectively, and the results showed that success rates and efficiency of linear hierarchical navigation were superior to hyperlinked navigation. In this study, using linear hierarchical navigation people have to follow steps without skipping, while using hyperlinked navigation people can directly search functions, jumping to the desired pages. Due to the unfamiliarity of smartphone operation, degenerated cognitive speed and memory loss, it was easier for the elderly to accept linear hierarchical navigation.

Positive affective experience variation was greatly influenced by navigation. NS together with CL had significant effects on positive affective experience variation, which may be because when readers cannot find the corresponding functions, disorientation will be increased, and when navigation effectively guided readers to find functions, readers will have positive affective experience such as joy, happiness, and confidence. As shown in Figure 2, when linear hierarchical navigation helped readers to find corresponding functions, positive affect was ascended obviously, and negative affect was slightly down. Readers may get lost in mixed navigation, which caused the significant decrease of positive affect and the slight increase of negative affect. Interaction effects of CL and NS results illustrated that two kinds of NSs in low cognitive load less affected affective experience, affective experience variation ranged in (−0.5, 0.5), but in high cognitive load condition, effects of two NSs on affective experience were amplified. Therefore, in the present study, NSs had great influence on affective experience, while CL had regulating effects in affective experience.

Usability analysis reported that NSs impacted on usability significantly. Score of linear hierarchical NS was higher than that of mixed NS. As previously mentioned, NS had played a very important role in information search. Well-designed NSs not only helped to improve usability, but also increased user’s positive affective experience. Therefore, the choice of NSs was meaningful to improve UX of digital reading for different populations.

In conclusion, NSs had significant effects on task completion time and positive affective experience variation, and interaction effects between CL and NS significantly influenced positive affect. NS significantly impacted on usability. Interaction effects between CL and NS were significant only on completion time and positive affective experience variation of task 1.

### Other Effects

As previously mentioned, correlations analysis examined usability did not significantly correlate with positive and negative affective variation, which suggested when NSs changed in two CL conditions in the present study, positive and negative affect will not synchronize significantly or reversely along with usability variation. But there were significantly negative correlations between positive and negative affective experience, which indicated that when positive affective experience increased, negative affective experience would decrease. In the domain of digital reading of smartphones, either promoting positive affective experience or reducing negative affective experience, will help improve the overall affective experience. Note: factors causing affective experience variation included the performing order of tasks. The current study used the same task order, which may also be one of the potential factors resulting in affective experience variation.

To summarize, when older adults search for information using linear hierarchical navigation, task efficiency, positive affective experience variation, and usability scores were significantly higher than using mixed navigation, which indicated that linear hierarchical navigation was more usable for the elderly. Linear hierarchical navigation is more appropriate when practitioners provide digital services for older adults.

However, there are limitations in our experiments. This study used two versions of existing websites which have different graphical treatment. It can be a confounded independent variable. Other design differences between the interfaces could have impacted the outcome. Therefore the result might be different. Future studies will refine the experiment to increase external validity. Researchers could build on this study by extending it to other information search tasks for older adults on smartphones. It would be both relevant and helpful.

## Conclusion

In this study, older adults performed information search tasks of NDLC using two NSs under different time pressures. Overall, NS had a significant impact on task performance and usability. Linear hierarchical navigation impacted the positive affective experience based on changes in CL. The results highlighted that the linear hierarchical NS is more appropriate for older people on smartphones. Information navigation patterns and contents should be built for different user groups, which will help to promote information navigation performance.

Cognitive load had slight effects on task performance, affective experience, and usability. However, when CL together with NS shifted, CL had a great influence on task performance and affective experience. CL is ubiquitous for different older adults. It is affected by many factors such as knowledge, experience, and time pressure. The results in this study showed that it played a role of accelerant. Therefore, it should be considered as a key design factor in human-computer interaction on smartphones.

This study only examined two types of navigation system in layout design. However, the design of online system navigations evolved with the development of information technology to serve the needs of users. In future studies, navigations systems and their application scenarios should be explored to find out how to design appropriately to satisfy older adults in different scenarios.

## Data Availability Statement

The raw data supporting the conclusions of this article will be made available by the authors, without undue reservation.

## Ethics Statement

Ethical review and approval was not required for the study on human participants in accordance with the local legislation and institutional requirements. The patients/participants provided their written informed consent to participate in this study.

## Author Contributions

AS contributed the idea of this study and wrote the most of the manuscript. FH in charge of recruting participants and expreiment design. DH reviewed the draft and made revisions. All authors contributed to the article and approved the submitted version.

## Conflict of Interest

The authors declare that the research was conducted in the absence of any commercial or financial relationships that could be construed as a potential conflict of interest.

## Publisher’s Note

All claims expressed in this article are solely those of the authors and do not necessarily represent those of their affiliated organizations, or those of the publisher, the editors and the reviewers. Any product that may be evaluated in this article, or claim that may be made by its manufacturer, is not guaranteed or endorsed by the publisher.
